# Relationship between sacroiliitis and inflammatory markers in familial Mediterranean fever

**DOI:** 10.1590/1806-9282.20240068

**Published:** 2024-05-20

**Authors:** Irfan Atik, Seda Atik

**Affiliations:** 1 Sivas Cumhuriyet University, Faculty of Medicine, Department of Radiology – Sivas, Turkey.; 2 Sivas Cumhuriyet University, Faculty of Medicine, Department of Physical Medicine and Rehabilitation, Division of Rheumatology – Sivas, Turkey.

**Keywords:** Familial Mediterranean fever, Inflammation, Sacroiliitis

## Abstract

**OBJECTIVE:**

Familial Mediterranean fever is the most common monogenic autoinflammatory disease. This study aimed to evaluate the relationship between sacroiliitis observed in familial Mediterranean fever and hematological inflammatory markers.

**METHODS:**

In this study, 168 familial Mediterranean fever patients were examined. A total of 61 familial Mediterranean fever patients who had sacroiliac magnetic resonance imaging due to waist and hip pain were included in the study. According to the magnetic resonance imaging findings, patients were divided into two groups: with and without sacroiliitis. The relationship between hematological inflammatory markers and sacroiliitis was investigated.

**RESULTS:**

The frequency of sacroiliitis was found to be 13.6% in all familial Mediterranean fever patients and 37.8% in patients with low back pain who underwent sacroiliac magnetic resonance imaging. Neutrophil count, neutrophil/lymphocyte ratio, monocyte/lymphocyte ratio, and systemic immune-inflammatory index were significantly higher in the sacroiliitis group than in the other group, and this difference was found to be statistically significant (p<0.05). As a result of the receiver operating characteristic analysis, it was observed that neutrophil/lymphocyte ratio, monocyte/lymphocyte ratio, and systemic immune-inflammatory index were very sensitive parameters in determining sacroiliitis in patients with familial Mediterranean fever.

**CONCLUSION:**

It was observed that the frequency of sacroiliitis was increased in familial Mediterranean fever patients. It is predicted that hematological inflammatory markers such as neutrophil/lymphocyte ratio, monocyte/lymphocyte ratio, and systemic immune-inflammatory index can be used in the diagnosis of sacroiliitis.

## INTRODUCTION

Familial Mediterranean fever (FMF) is the most common monogenic autoinflammatory disease. It is characterized by peritonitis, pleuritis, and acute synovitis attacks and is the prototype of relapsing fever syndromes^1^. It is more common in populations of Mediterranean origin such as Arabs, Turks, Jews, and Armenians. Acute relapsing arthritis is the most common form of musculoskeletal involvement in FMF. However, chronic arthritis, including sacroiliitis, may develop in ≤5% of FMF patients^2^. Studies conducted on Turkish FMF patients have reported a high frequency of sacroiliitis^3-5^.

The MEFV gene, which is mapped on the short arm of chromosome 16, is associated only with FMF. The most common MEFV mutations among Arabs, Turks, Jews, and Iranians are M694V, E148Q, M680I, and V726A. These genes differ in terms of penetrance and correlation with the severity of clinical symptoms^6^.

Inflammation: In many diseases, especially rheumatological diseases, it was found to be related to the severity, clinical presentation, and prognosis of the disease. Various ratios are employed to determine the level of inflammation, which are considered to be superior to the quantitative count of white blood cells. These ratios, which are obtained by dividing cell counts, include the neutrophil/lymphocyte ratio (NLR), platelet/lymphocyte ratio (PLR), and monocyte/lymphocyte ratio (MLR), as well as the systemic immune-inflammatory index (SII)^7-9^. NLR is a diagnostic inflammatory marker studied in gastrointestinal^10-12^, endocrine^13^, cardiac^14^, and infectious^15^ diseases. Similarly, MLR is another inflammatory marker studied in malignancies^16^, gastrointestinal diseases^12^, and endocrine pathologies such as diabetes mellitus^17^, showing its significance. SII, which is another inflammation marker, has been investigated for its diagnostic value in various diseases, including sacroiliitis, and is a valuable indicator^18,19^. FMF is an autoinflammatory rheumatic disease, and sacroiliitis can be observed in FMF patients as it is associated with inflammation^20^.

This study aimed to investigate the presence of sacroiliitis in FMF patients, the relationship between sacroiliitis and gene mutation, and the diagnostic value of inflammatory markers in FMF patients with sacroiliitis.

## METHODS

This is a single-center, retrospective, cross-sectional study. Approval for the study was received from the local university ethics committee. Patients who were previously diagnosed with FMF by Tel-Hashomer criteria had low back and hip pain and underwent sacroiliac magnetic resonance imaging (MRI) in the Department of Radiology between January 2018 and October 2023 were included in the study. All MRIs were prospectively reinterpreted by a musculoskeletal radiologist and rheumatologist blinded to the patient's clinical status. Evaluations were made according to the Assessment of SpondyloArthritis International Society (ASAS) MRI working group definition of active sacroiliitis, and erosions, subchondral bone edema, and synovitis were findings that suggested the diagnosis of sacroiliitis^21^. According to the Declaration of Helsinki, the rights of all participants were protected. Coexistence of FMF and spondyloarthritis, psoriasis, recurrent oral-genital aphtha, inflammatory bowel disease, and amyloidosis were determined as exclusion criteria.

The patient's age, gender, genetic test results, and laboratory test results such as neutrophils, lymphocytes, monocytes, platelets, sedimentation, and C-reactive protein were recorded. NLR, MLR, and SII were used to evaluate the inflammation level. NLR and MLR levels were obtained by dividing the values measured in the complete blood count. SII value was calculated according to the formula platelet ´ neutrophil/lymphocyte ratio.

### Statistical analysis

The data from the study were analyzed using the Statistical Package for Social Sciences (SPSS) version 22.0. The normality of the variables was assessed using the Kolmogorov-Smirnov test. For continuous variables that followed a normal distribution, the mean±standard deviation (SD) was reported, while for non-normally distributed variables, the median and minimum-maximum values were provided. Categorical data were presented in terms of frequency and percentage. To compare normally distributed continuous variables, the independent-sample t-test was used, and for non-normally distributed continuous variables, the Mann-Whitney U test was employed. Qualitative data analysis was performed using the chi-square (χ^2^) test. In evaluating some quantitative data, diagnostic performance (DP) was determined through receiver operating characteristic (ROC) analysis. The significance level for statistical tests was set at 0.05.

## RESULTS

Between January 2018 and October 2023, 168 FMF patients were scanned from medical records. A total of 61 FMF patients who had sacroiliac MRI and met the inclusion and exclusion criteria were evaluated. These patients were divided into two groups, with and without sacroiliitis, according to the available MRI findings. The frequency of sacroiliitis was found to be 13.6% in the screened FMF cohort and 37.8% in patients with low back pain who underwent sacroiliac MRI. The demographic characteristics and laboratory findings of the patients are presented in comparative detail in Table 1.

**Table 1:** Comparison of demographic and laboratory findings of the study group and neutrophil/lymphocyte ratio, monocyte/lymphocyte ratio, and systemic immune-inflammatory index values.

Gender (n, %)	Sacroiliitis (+)	Sacroiliitis (-)	p
Female	14 (23%)	27 (44.2%)	
Male	9 (14.8%)	11 (18%)	0.41^a^
Age, years (mean ± SD)	36.60±12.14	37.73±11.36	0.71^b^
CRP, mg/L median (min–max)	3.28 (0.66–6.90)	2.56 (0.21–7.58)	0.30^c^
ESR, mm/h median (min–max)	11 (2–34)	11.5 (2–39)	0.74^c^
MPV, fl (mean ± SD)	10.04±0.97	10.20±0.84	0.50^b^
Neutrophil count (10^9^/L) median (min–max)	4.52 (2.95–12.70)	3.41 (1.27–6.49)	**0.001** * c
Lymphocyte count (10^9^/L) median (min–max)	2.19 (1.32–3.88)	2.51 (1.15–5.73)	0.23^c^
Monocyte count (10^9^/L) median (min–max)	0.5 (0.27–1.30)	0.42 (0.22–1.21)	0.11^c^
Platelet count (10^9^/L) median (min–max)	293 (208–645)	273 (175–526)	0.12^c^
NLR [median (min–max)]	2.01 (1.40–4.35)	1.38 (0.73–3.61)	**0.0001** * c
MLR [median (min–max)]	0.24 (0.11–0.47)	0.18 (0.09–0.57)	**0.009** * c
SII [median (min–max)]	738.63 (409.15–1239.55)	387.72 (190.0–987.92)	**0.0001** * c

Statistically significant values are indicated in bold. CRP: C-reactive protein; ESR: erythrocyte sedimentation rate; MPV: mean platelet volume; NLR: neutrophil/lymphocyte ratio; MLR: monocyte/lymphocyte ratio; SII: systemic immune-inflammatory index.

* p<0.05: statistically significant.

a Chi-square analysis was used.

b Independent-sample t-test was used.

c Mann-Whitney U test was used.

**Figure 1:**
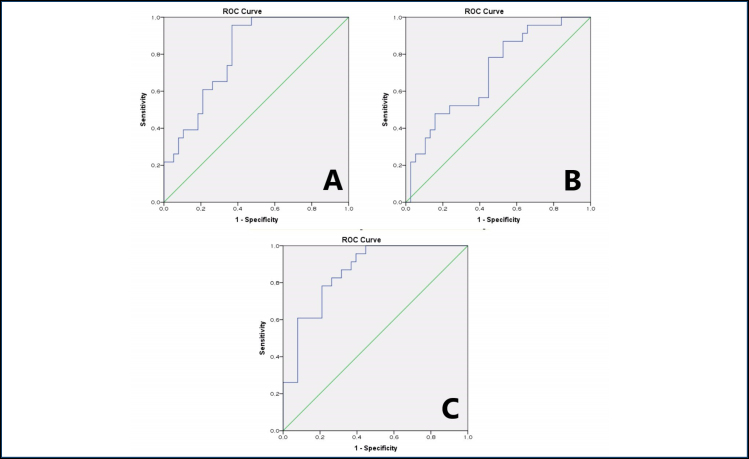
(A) Receiver operating characteristic curve for neutrophil/lymphocyte ratio measurement according to the presence of sacroiliitis in patients with familial Mediterranean fever (AUC: 0.801). (B) Receiver operating characteristic curve for monocyte/lymphocyte ratio measurement according to the presence of sacroiliitis in patients with familial Mediterranean fever (AUC: 0.700). (C) Receiver operating characteristic curve for systemic immune-inflammatory index measurement according to the presence of sacroiliitis in patients with familial Mediterranean fever (AUC: 0.858).

A significant difference was detected in the NLR, MLR, and SII values in the group with sacroiliitis compared with the other group. Based on this result, the ROC curve was used to evaluate the cutoff values, sensitivity, and specificity in terms of the importance of NLR, MLR, and SII in the development of sacroiliitis in patients with FMF (Table 2 and Figure 1).

**Table 2:** Diagnostic screening tests for neutrophil/lymphocyte ratio, monocyte/lymphocyte ratio, and systemic immune-inflammatory index based on the presence of sacroiliitis and receiver operating characteristic curve results.

	Diagnostic scan			ROC curve		p
	Cutoff	Sensitivity	Specificity	Area	95% confidence interval	
NLR	≥ ***1.70***	73.90	65.80	**0.801**	0.693–0.908	***0.0001*** *
MLR	≥ ***0.193***	78.30	55.30	**0.700**	0.567–0.833	***0.009*** *
SII	≥ ***524.07***	78.30	78.90	**0.858**	0.767–0.949	***0.0001*** *

Statistically significant values are indicated in bold or italic. NLR: neutrophil/lymphocyte ratio; MLR: monocyte/lymphocyte ratio; SII: systemic immune-inflammatory index.

* p<0.05: statistically significant.

Genetic analysis results of the patients included in the study were as follows: homozygous M694V (M694V/MV94V) in 13 patients (21.3%), heterozygous M694V (M694V/-) in 10 patients (16.3%), heterozygous R202Q (R202Q/-) in 7 patients (11.4%), heterozygous E148Q (E148Q/-) in 7 patients (11.4%), homozygous V726A (V726A/V726A) in 6 patients (9.8%), combined heterozygous M694V/M680I in 5 patients (8.2%), heterozygous M680I (M680I/-) in 4 patients (6.5%), homozygous M680I (M680I/M680I) in 3 patients (5.1%), heterozygous A744S (A744S/-) in 2 patients (3.2%), heterozygous P369S (P369S/-) in 1 patient (1.7%), heterozygous PGLN1678 (PGLN1678/-) in 1 patient (1.7%), homozygous R202Q (R202Q/R202Q) (1.7%) in 1 patient, and heterozygous V722M (V722M/-) in 1 patient (1.7%).

As the majority of patients had the M694V mutation, the relationship between this genotype and sacroiliitis was evaluated. The result was found to be statistically significant (p<0.05).

## DISCUSSION

Although sacroiliitis is generally known as a distinguishing feature of spondyloarthropathies, it is also observed with increasing frequency in Turkish and Jewish FMF patients. In the study conducted by Cefle et al. in Turkish FMF patients, the frequency of sacroiliitis was found to be 10.5%, and in the study by Kaşifoğlu et al. it was found to be 7% in all FMF patients and 32.7% in those with complaints^3,5^. Similarly, in this study, the frequency of sacroiliitis was 13.6% in all FMF patients and 37.8% in those with low back pain. Yildiz et al. found that the rate was found to be 50% in cases in which sacroiliitis was evaluated with Tc99m-MDP bone scintigraphy^4^. It is thought that the differences in the results of many studies may be due to changes such as differences in the patient population and radiological screening methods.

Many studies have been conducted showing that FMF involvement may be associated with different genotypes. M694V mutation is the most common mutation known to be associated with severe disease in Turkish patients^22^. Many studies have shown that the M694V mutation is associated with many different severe phenotypes, such as earlier disease onset, more frequent attacks, higher prevalence of arthritis, pleuritis, erysipelas-like erythema, requirement for high doses of colchicine, and increased risk of amyloidosis. In this study, the risk of developing sacroiliitis in patients with the M694V mutation was found to be statistically significantly higher than in those without it. Gene mutation results of FMF patients who were not included in the study are unknown. This result may not be generalizable to all FMF patients. This is among the limitations of this study.

Another aim of this study was to evaluate subclinical inflammation in the attack-free period in FMF patients who develop sacroiliitis and to determine its contribution to the development of sacroiliitis. To determine subclinical inflammation, NLR, MLR, and SII were used, which have been used in many studies before and are considered to guide in the progression, diagnosis, and treatment of diseases. We found these values to be significantly higher in FMF patients with sacroiliitis compared with those without. Kelesoglu et al. evaluated subclinical inflammation in pediatric FMF patients according to mutation type and attack status and found the CRP level in patients with M694V mutation to be significantly different compared with other mutations^23^.

Neutrophil/lymphocyte ratio, PLR, mean platelet volume (MPV), and red cell width distribution (RDW) were used in the study by Özer et al. to determine a new inflammatory marker to indicate subclinical inflammation in FMF patients. While it was concluded that all of them could be used to determine subclinical inflammation, the most powerful marker was NLR^24^. In this study, markers such as NLR, MLR, and SII were used, whose relationship with sacroiliitis in FMF patients has not been investigated before. Additionally, we did not find any other study in the literature that investigated the role of SII and its relationship with the disease in FMF patients. We concluded that NLR, PLR, and SII are very valuable markers in predicting sacroiliitis in patients with FMF. In addition, the cutoff values are found with ROC analysis and are intended to be easily used daily.

Magnetic resonance imaging is the most sensitive method recommended for detecting sacroiliitis^25^. However, MRI is an imaging method that is not available in every center and is costly. Considering these factors, we believe that inflammatory markers may contribute to the diagnosis and at least guide first-line treatment in patients with symptoms.

The positive aspects of this study are the evaluation of the usability of NLR, MLR, and SII, whose relationship with sacroiliitis in FMF patients has not been studied before, in the diagnosis of sacroiliitis and the simple evaluation and conclusion of the results with hemogram examination, which can be used frequently in many patients. There are some limitations in this study. This study was designed retrospectively, which may cause selection bias. Another limitation is that the relationship between mutations and sacroiliitis could not be evaluated due to the lack of sufficient patients in all genotype groups. Studies with larger numbers of patients are needed. We believe that the long-term results of the association of FMF and sacroiliitis can be evaluated by long-term follow-up of these patients.

## CONCLUSION

The frequency of sacroiliitis is increased in FMF patients. Inflammatory markers such as NLR, MLR, and SII can be used in clinical practice to predict sacroiliitis in patients with symptoms.
